# Prognostic significance of the combination of preoperative hemoglobin and albumin levels and lymphocyte and platelet counts (HALP) in patients with renal cell carcinoma after nephrectomy

**DOI:** 10.1186/s12894-018-0333-8

**Published:** 2018-03-15

**Authors:** Ding Peng, Cui-jian Zhang, Qi Tang, Lei Zhang, Kai-wei Yang, Xiao-teng Yu, Yanqing Gong, Xue-song Li, Zhi-song He, Li-qun Zhou

**Affiliations:** 1 Department of Urology, Peking University First Hospital, No. 8, Xishiku Street, Xicheng District, Beijing, 100034 China; 2 Institute of Urology, Peking University, Beijing, 100034 China; 3 National Urological Cancer Center, Beijing, 100034 China; 4 Urogenital Diseases (male) Molecular Diagnosis and Treatment Center, Peking University, Beijing, 100034 China

**Keywords:** Renal cell carcinoma, Prognosis, HALP, Nephrectomy

## Abstract

**Background:**

To evaluate the prognostic significance of the novel index combining preoperative hemoglobin and albumin levels and lymphocyte and platelet counts (HALP) in renal cell carcinoma (RCC) patients.

**Methods:**

We enrolled 1360 patients who underwent nephrectomy in our institution from 2001 to 2010. The cutoff values for HALP, neutrophil-to-lymphocyte ratio and platelet-to-lymphocyte ratio were defined by using X-tile software. Survival was analyzed by the Kaplan–Meier method, with differences analyzed by the log-rank test. Multivariate Cox proportional-hazards model was used to evaluate the prognostic significance of HALP for RCC.

**Results:**

Low HALP was significantly associated with worse clinicopathologic features. Kaplan-Meier and log-rank tests revealed that HALP was strongly correlated with cancer specific survival (*P* < 0.001) and Cox multivariate analysis demonstrated that preoperative HALP was independent prognostic factor for cancer specific survival (HR = 1.838, 95%CI:1.260–2.681, *P* = 0.002). On predicting prognosis by nomogram, the risk model including TNM stage, Fuhrman grade and HALP score was more accurate than only use of TNM staging.

**Conclusions:**

HALP was closely associated with clinicopathologic features and was an independent prognostic factor of cancer-specific survival for RCC patients undergoing nephrectomy. A nomogram based on HALP could accurately predict prognosis of RCC.

**Electronic supplementary material:**

The online version of this article (10.1186/s12894-018-0333-8) contains supplementary material, which is available to authorized users.

## Background

Renal cancer accounts for 2% to 3% of all cancers, and the rate of renal cell carcinoma (RCC) has increased by 1.6% per year for the past 10 years [1]. Approximately 90% of renal cancer is RCC, and surgery is the only curative treatment. About 20% of RCC patients have advanced stage disease, and for those with localized RCC, nearly 30% show recurrence after tumor resection [2, 3]. Therefore, we need better prognostic models to improve prognosis.

The TNM stage, reflecting tumor invasion, lymph node metastasis and distant metastasis, is the most widely used system for predicting RCC prognosis [2]. However, because of heterogeneous prognoses, the outcomes of some patients with the same stage of cancer may be completely different. Therefore, we need useful biomarkers to increase the prognostic accuracy in RCC.

Increasing evidence supports that inflammation and nutrition are involved in the initiation and progression of various cancers, including RCC [4]. Hematologic parameters including albumin and hemoglobin levels and lymphocytes, neutrophils and platelets counts are easily acquired laboratory data reflecting inflammation and nutrition status and have been extensively studied. Numerous studies have reported the prognostic value of serum albumin and hemoglobin levels and lymphocyte and platelet counts for various cancers, including RCC [5–8]. However, the disadvantage of these indicators is that each reflects only one respect of inflammation or nutrition. Further studies found that the combination of those factors in an index such as the prognostic nutritional index (PNI), combining albumin level and lymphocyte count, or the neutrophil-to-lymphocyte ratio (NLR), lymphocyte-to-monocyte ratio (LMR) or platelet-to-lymphocyte ratio (PLR) could more accurately predict prognosis than a single index [9–12].

A novel index combining hemoglobin and albumin levels and lymphocyte and platelet counts (HALP) has been found significantly associated with outcomes in colorectal and gastric cancer [13, 14]. In this study, we investigated the clinical value of this index in RCC patients undergoing nephrectomy.

## Methods

### Patients

We included 1360 patients with histologically confirmed RCC. All patients were underwent nephrectomy in the Department of Urology, Peking University First Hospital, between 2001 and 2010. Clinicopathologic characteristics and laboratory data were collected. Follow-up care including abdominal ultrasonography or abdominal CT, chest X-ray, and laboratory tests was performed at regular intervals (3-month intervals in years 1 to 3, 6-month intervals in years 4 to 5, and 12-month intervals in years 6 to 10 after diagnosis).

### Statistical analysis

Data are presented as number (percentage) for categorical variables and median (interquartile range [IQR]) for continuous variables. HALP was calculated as hemoglobin level (g/L) × albumin level (g/L) × lymphocyte(/L)/platelet count (/L), NLR as neutrophil-to-lymphocyte count and PLR as platelet-to-lymphocyte count. The cut-off values for NLR, PLR and HALP were determined by using X-tile v3.6.1 (Yale University) [15]. The X-tile software was able to compare the *P* values of different cut-off values for a continuous variable and determine the best cut-off value with the most significant P value. Chi-square test was used to analyze an association of clinicopathologic data with HALP. The Kaplan-Meier survival method was used to estimate cancer-specific survival (CSS), with log-rank test used to test significant differences. The significant variables in the univariate analysis were included in the Cox proportional-hazards regression multivariate survival analyses by Forward LR method. Statistical analyses involved use of SPSS v22.0 (SPSS Inc., Chicago, IL, USA) and *P* < 0.05 was considered statistically significant.

## Results

### Patient characteristics

We included 1360 patients (952 men, median age 55 years [IQR 46–65]) (Table 1). The median follow-up was 67 months (IQR 36–74) and 139 (10.2%) patients died due to RCC during follow-up. The 5-year estimated CSS was 89.4% for all patients.

**Table 1:** Basline clinicopathologic characteristics of 1360 patients with renal cell carcinoma (RCC) undergoing nephrectomy

Characteristics	Total *n* = 1360
Age, years, median (IQR)	55 (46–65)
Female sex	408 (30%)
Histology subtype	
ccRCC	1228 (90.29%)
non-ccRCC	132 (9.71%)
Location	
left	626 (46.03%)
right	697 (51.25%)
bilateral	37 (2.72%)
Fuhrman grade	
1	374 (27.5%)
2	738 (54.26%)
3	237 (17.43%)
4	11 (0.81%)
T-stage	
1	1015 (74.63%)
2	113 (8.32%)
3	225 (16.54%)
4	7 (0.51%)
N status	
negative	1327 (97.57%)
positive	33 (2.43%)
ASA grade	
1	192 (14.12%)
2	1072 (78.82%)
3&4	96 (7.06%)
Sarcomatoid transformation	65 (4.78%)
Metastasis	61 (4.48%)
Lymphovascular invasion	100 (7.35%)
Necrosis	419 (3.08%)
Hypoalbuminemia	53 (3.9%)
Anemia	267 (19.6%)
NLR, median (IQR)	2.13 (1.60–2.85)
PLR, median (IQR)	124.07 (97.33–165.22)
HALP, median (IQR)	47.48 (33.21–63.45)

Data are n (%) unless indicated. *IQR* interquartile ratio, *ccRCC* clear-cell renal cell carcinoma, *ASA* American Society of Anesthesiologists, *NLR* neutrophil-to-lymphocyte ratio, *PLR* platelet-to-lymphocyte ratio, *HALP* hemoglobin and albumin levels and lymphocyte and platelet counts

### Association of HALP and clinicopathologic features

**Fig. 1:**
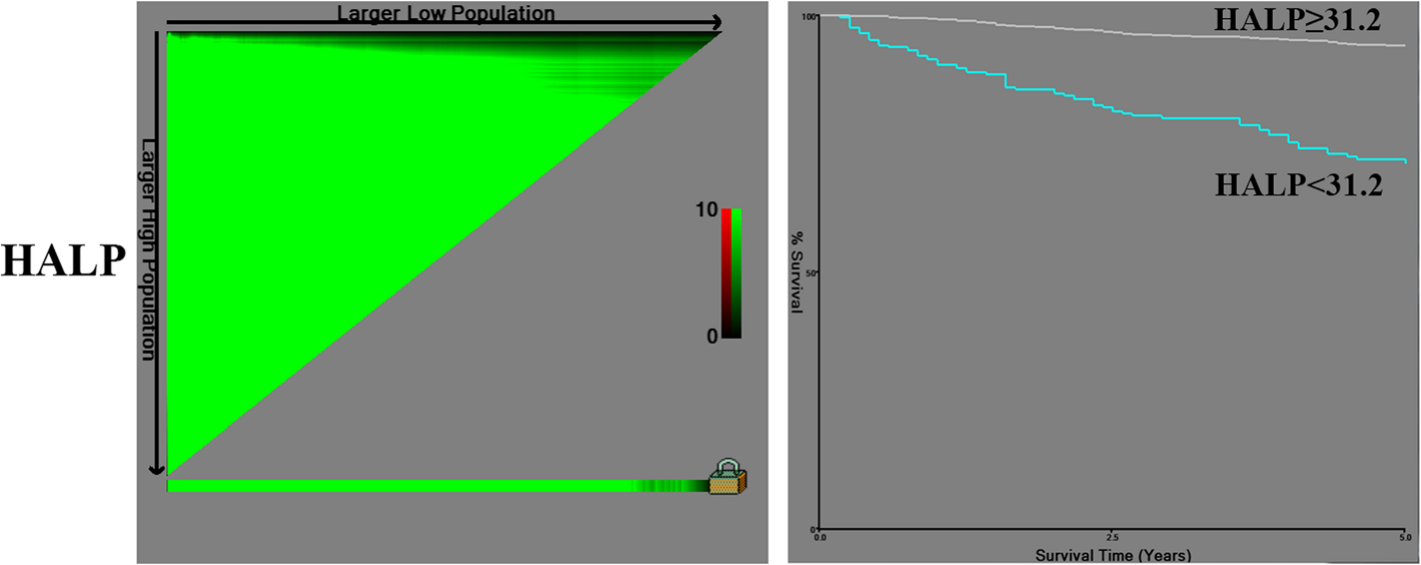
Cut off value for hemoglobin and albumin levels and lymphocyte and platelet counts (HALP) by using X-tile

We detected cut-off values for HALP, 31.2; NLR, 2.9; and PLR, 198.3 (Fig. 1 and Additional file 1: Figure S1) for dividing patients into low and high HALP, NLR and PLR groups. Decreased HALP level was associated with being female, older age, high Fuhrman grade and high T stage and N and M positive status, sarcomatoid transformation, tumor necrosis, lymphovascular invasion and low NLR or PLR (Table 2).

**Table 2:** Association of baseline clinicopathologic characteristics and HALP

Variable	*n* (%)		HALP^a^	
		low (%)	high (%)	*P* value
All patients	1360	291 (21.40%)	1069 (78.60%)	
Gender				< 0.001
male	952 (70.00%)	162 (17.02%)	790 (82.98%)	
female	408 (30.00%)	129 (31.62%)	279 (68.38%)	
Age, years				< 0.001
≤ 65	997 (73.31%)	186 (18.66%)	811 (81.34%)	
>65	363 (26.69%)	105 (28.93%)	258 (71.07%)	
Histology subtype				0.695
ccRCC	1228 (90.29%)	261 (21.25%)	967 (78.75%)	
non-ccRCC	132 (9.71%)	30 (22.73%)	102 (77.27%)	
ASA grade				0.361
1 + 2	1264 (92.94%)	268 (21.20%)	996 (78.80%)	
3 + 4	96 (7.06%)	23 (23.96%)	68 (76.04%)	
Fuhrman grade				< 0.001
1 + 2	1112 (81.76%)	177 (15.92%)	935 (84.08%)	
3 + 4	248 (18.24%)	113 (45.56%)	130 (54.44%)	
T stage				< 0.001
1 + 2	1128 (82.94%)	173 (15.34%)	955 (84.66%)	
3 + 4	232 (17.06%)	118 (50.86%)	113 (49.14%)	
N status				< 0.001
negative	1327 (97.57%)	270 (20.35%)	1057 (79.65%)	
positive	33 (2.43%)	21 (63.64%)	12 (36.36%)	
Metastasis				< 0.001
negative	1299 (95.51%)	258 (19.86%)	1041 (80.14%)	
positive	61 (4.49%)	33 (54.10%)	28 (45.90%)	
Sarcomatoid transformation				< 0.001
absent	1295 (95.22%)	245 (18.92%)	1050 (81.08%)	
present	65 (4.78%)	46 (70.77%)	19 (29.23%)	
Tumor necrosis				< 0.001
absent	940 (69.12%)	158 (16.81%)	782 (83.19%)	
present	420 (30.88%)	133 (31.67%)	286 (68.33%)	
Lymphovascular invasion				< 0.001
absent	1260 (92.65%)	249 (19.76%)	1011 (80.24%)	
present	100 (7.35%)	42 (42%)	58 (58%)	
NLR				< 0.001
high	317 (23.3%)	166 (52.37%)	151 (47.63%)	
low	1043 (76.7%)	125 (11.98%)	918 (88.02%)	
PLR				< 0.001
high	195 (14.3%)	180 (92.31%)	15 (7.69%)	
low	1165 (85.7%)	111 (9.53%)	1054 (90.47%)	

^a^data are number of patients for those with HALP < and ≥ 31.2

### Association of HALP with patient outcomes

**Fig. 2:**
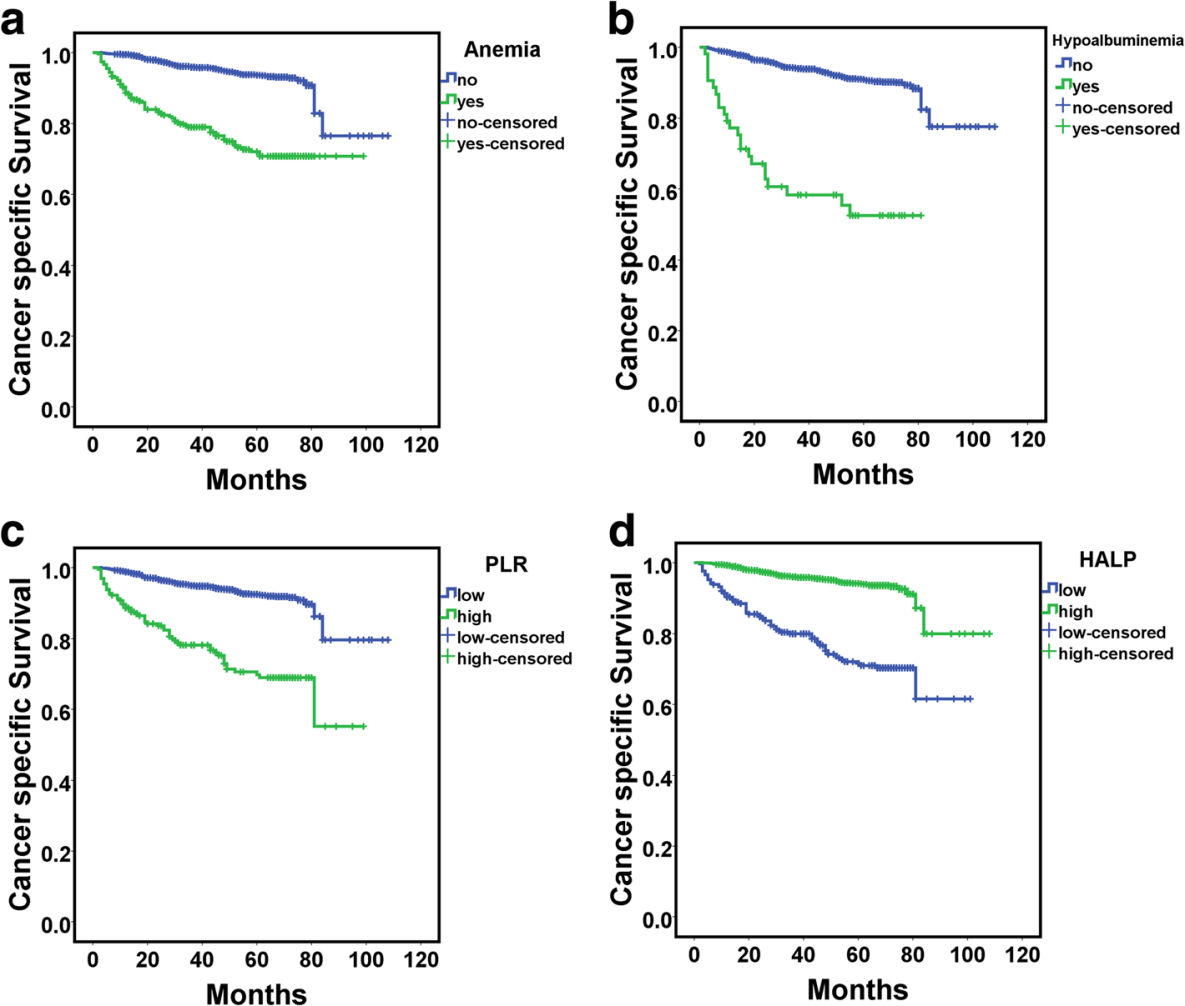
Kaplan-Meier curves for cancer-specific survival in patients with RCC according to anemia (**a**), hypoalbuminemia (**b**), platelet-to-lymphocyte ratio (PLR)(**c**) and hemoglobin and albumin levels and lymphocyte and platelet counts (HALP) (**d**)

On univariate analysis, all included clinicopathologic features except for age (*P* = 0.287), gender (*P* = 0.226), histology subtype (*P* = 0.385) and American Society of Anesthesiologists grade (*P* = 0.964) were significantly related to survival outcomes (Table 3). Anemia and hypoalbuminemia, high PLR and low HALP were all significantly associated with worse survival (Fig. 2). On multivariate analyses, prognostic factors for CSS with RCC were Fuhrman grade (HR 1.767, 95% CI 1.177–2.652, *P* = 0.006), T stage (3.890, 2.510–6.030, *P* < 0.001), N stage (2.480, 1.526–4.032, *P* < 0.001), M stage (4.728, 3.090–7.233, *P* < 0.001) and HALP (1.838, 1.260–2.681, *P* = 0.002) (Table 3).

**Table 3:** Univariate and multivariate analyses of factors associated with cancer-specific survival for RCC patients

Variable	Univariate analysis *P*	Multivariate analysis	
		HR (95% CI)	*P*
Age (>65 vs ≤65)	0.287		
Gender (female vs male)	0.226		
Histology subtype (non-ccRCC vs ccRCC)	0.385		
ASA grade (3 + 4 vs 1 + 2)	0.964		
Fuhrman grade (3 + 4 vs 1 + 2)	< 0.001	1.767 (1.177–2.652)	0.006
T stage (3 + 4 vs 1 + 2)	< 0.001	3.890 (2.510–6.030)	< 0.001
N status (positive/negative)	< 0.001	2.480 (1.526–4.032)	< 0.001
M status (positive vs negative)	< 0.001	4.728 (3.090–7.233)	< 0.001
Sarcomatous differentiation (present vs absent)	< 0.001		
Lymphovascular invasion (present vs absent)	< 0.001		
Necrosis (present vs absent)	< 0.001		
Hypoalbuminemia (present vs absent)	< 0.001		
Anemia (present vs absent)	< 0.001		
NLR (high vs low)	< 0.001		
PLR (high vs low)	< 0.001		
HALP (low vs high)	< 0.001	1.838 (1.260–2.681)	0.002

### Nomogram of HALP-based risk model for RCC

**Fig. 3:**
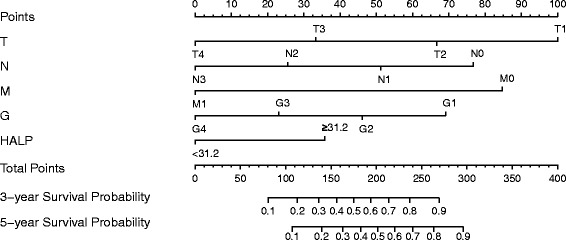
Nomogram for 3-year and 5-year survival with RCC

We next used nomogram to predict 3- and 5-year CSS for individual patients. Independent prognostic factors in the multivariate analysis including Fuhrman grade, TNM status and HALP were included in the nomogram (Fig. 3). Similar to multivariate findings, with nomogram, high Fuhrman grade and advanced TNM status were associated with poor prognosis and high HALP with favorable prognosis.

**Fig. 4:**
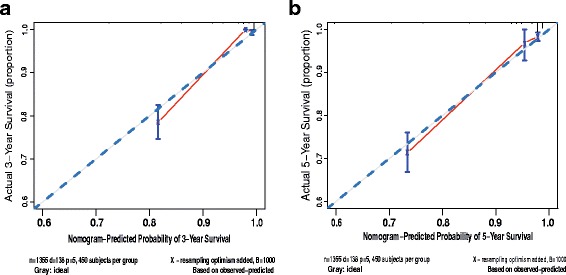
Calibration curve for 3-year (**a**) and 5-year (**b**) survival

The calibration curves of the nomogram showed that the predictive probability of 3- and 5-year survival was closely related to the actual 3- and 5-year survival (Fig. 4). The C-index was 0.881 (95% CI: 0.853–0.909) by this nomogram compared with 0.846 (0.812–0.880) with the TNM staging system. Hence, the risk model including TNM stage, Fuhrman grade and HALP had better prognostic prediction accuracy than the only TNM system.

## Discussion

In this study, we evaluated the prognostic significance of the novel index HALP combining hemoglobin and albumin levels and lymphocyte and platelet counts in RCC patients undergoing nephrectomy. HALP was closely associated with clinicopathologic features. Univariate and multivariate analyses demonstrated that HALP was an independent predictor of CSS for RCC patients undergoing nephrectomy. Furthermore, the nomogram based on HALP could predict prognosis more accurately than the TNM system.

There are several known predictive models of RCC such as TNM stage and the Stage, Size, Grading and Necrosis (SSIGN) model [16]. Inflammatory and nutritional indicators based on hematologic parameters such as albumin and hemoglobin levels and lymphocyte, neutrophil and platelet counts were also associated with outcomes with RCC. Moreover, several indicators combined with hematologic parameters, including NLR, LMR, and PLR, were more accurate predictors [6, 9, 10, 12]. Recently, indicators combining albumin level with LMR or NLR were found significantly associated with outcomes [17, 18], which suggests better prediction of outcomes by combining inflammatory and nutritional indicators.

Accumulating evidence suggests the important role of the inflammatory response and nutritional status in cancer progression and metastasis. Overall, 30% of cancer patients were found with cancer-related anemia (CRA) at the time of diagnosis and CRA was associated with more advanced cancer stage [19]. CRA is believed to be associated with chronic blood loss, iron deficiency, and vitamin B12 or folate nutritional deficiency. Meanwhile, imbalanced inflammation regulation, such as increased hepcidin and reactive oxygen species stress, in cancer patients is also responsible for CRA [20]. Serum albumin is synthesized specially in the liver and known as a negative acute-phase protein. In addition, systemic factors such as inflammation and stress could affect serum albumin level. Therefore, decreased serum albumin level represents a malnutrition status and also a sustained systemic inflammation response. As important indicators of nutrition and inflammation, anemia and hypoalbuminemia are widely reported to be associated with worse outcomes in various cancers including RCC [7, 8]. Morgan et al. [21] reported that for locoregional RCC patients undergoing nephrectomy, 25% of patients have anemia and 5.1% have hypoalbuminemia. The authors also found hypoalbuminemia (< 35 g/L), unintentional preoperative weight loss ≥5% and preoperative BMI < 18.5 kg/m2 as reflecting nutritional deficiency (ND) and that anemia and ND were independent predictors of overall mortality and disease-specific mortality. Preoperative hypoalbuminemia and anemia were also found to predict transfusion during radical nephrectomy for RCC [5]. We observed 3.9% hypoalbuminemia and 19.6% anemia in our patients, which is consistent with previous study. On univariate analysis, both anemia and hypoalbuminemia were associated with worse survival.

Cancer-related inflammation is considered the seventh hallmark of cancer, playing a conflicting role in tumor initiation and progression in that both tumor-antagonizing and -promoting leukocytes can be found [22]. Elevated neutrophil count is associated with cytokine secretion and contributes to tumor angiogenesis, promotion and metastasis. CD4+ and CD8+ T lymphocytes can enhance cancer immune-surveillance to inhibit tumour cell proliferation, invasion and metastasis [23]. Increased neutrophil count and decreased lymphocyte count might be responsible for a weak and insufficient immune response to tumors and strongly associated with a poor survival in advanced cancer [24, 25].

Recent data implied that the activation of platelets is crucial for cancer progression by promoting angiogenesis, extracellular matrix degradation, and release of growth factors, which are essential components of tumor growth and metastatic [26]. In addition, platelets adhering to tumor cells could secrete vascular endothelial growth factor(VEGF), which induces microvessel permeability, promotes extravasation of cancer cells, and induces neoangiogenesis [27]. Platelet count was found a significant predictor of RCC-specific mortality [28]. Recently, tumor-educated blood platelets (TEPs) were implicated as central players in the systemic and local responses to tumor growth. As well, the RNA profile in TEPs could provide a valuable platform for liquid biopsy [29].

Systemic inflammation markers including NLR, LMR, and PLR have been found associated with survival in many solid tumors including RCC [6, 10, 30, 31]. Among those indicators, the finding of elevated NLR and PLR indicated increased neutrophil and platelet count and decreased lymphocyte count associated with worse outcomes. In our study, elevated NLR and PLR were associated with worse CSS on univariate analysis. However, on multivariate analyses, HALP rather than NLR and PLR remained an independent prognostic factor.

We used nomogram of independent prognostic factors found on multivariate analyses including HALP and evaluated their accuracy by calibration curves. The predictive accuracy was better with HALP than the TNM system. TNM stage may be an important factor in RCC, but other factors such as HALP could be included and improve the prediction of outcomes.

The major limitations of the present study are its retrospective nature and the single-center design. Additional large and prospective studies are needed to confirm these findings.

## Conclusions

HALP was closely associated with clinicopathologic features of RCC patients undergoing nephrectomy and was an independent prognostic factor of CSS. A nomogram based on HALP could accurately predict prognosis with RCC. Preoperative HALP could be a novel indicator to evaluate the outcome for RCC patients after nephrectomy.

## Additional file

Additional file 1: **Figure S1.** Cut-off values for neutrophil-to-lymphocyte ratio (NLR) and platelet-to-lymphocyte ratio (PLR). (TIFF 6359 kb)

## Abbreviations

CRA: cancer-related anemia
CSS: cancer-specific survival
LMR: lymphocyte-to-monocyte ratio
NLR: neutrophil-to-lymphocyte ratio
PLR: platelet-to-lymphocyte ratio
PNI: prognostic nutritional index
RCC: renal cell carcinoma
