# Supplementary figures and images for: Prognostic significance of the combination of preoperative hemoglobin and albumin levels and lymphocyte and platelet counts (HALP) in patients with renal cell carcinoma after nephrectomy
Source: BMC Urol. 2018 Mar 15;18:20. doi: 10.1186/s12894-018-0333-8 (PMC5855974; doi:10.1186/s12894-018-0333-8)

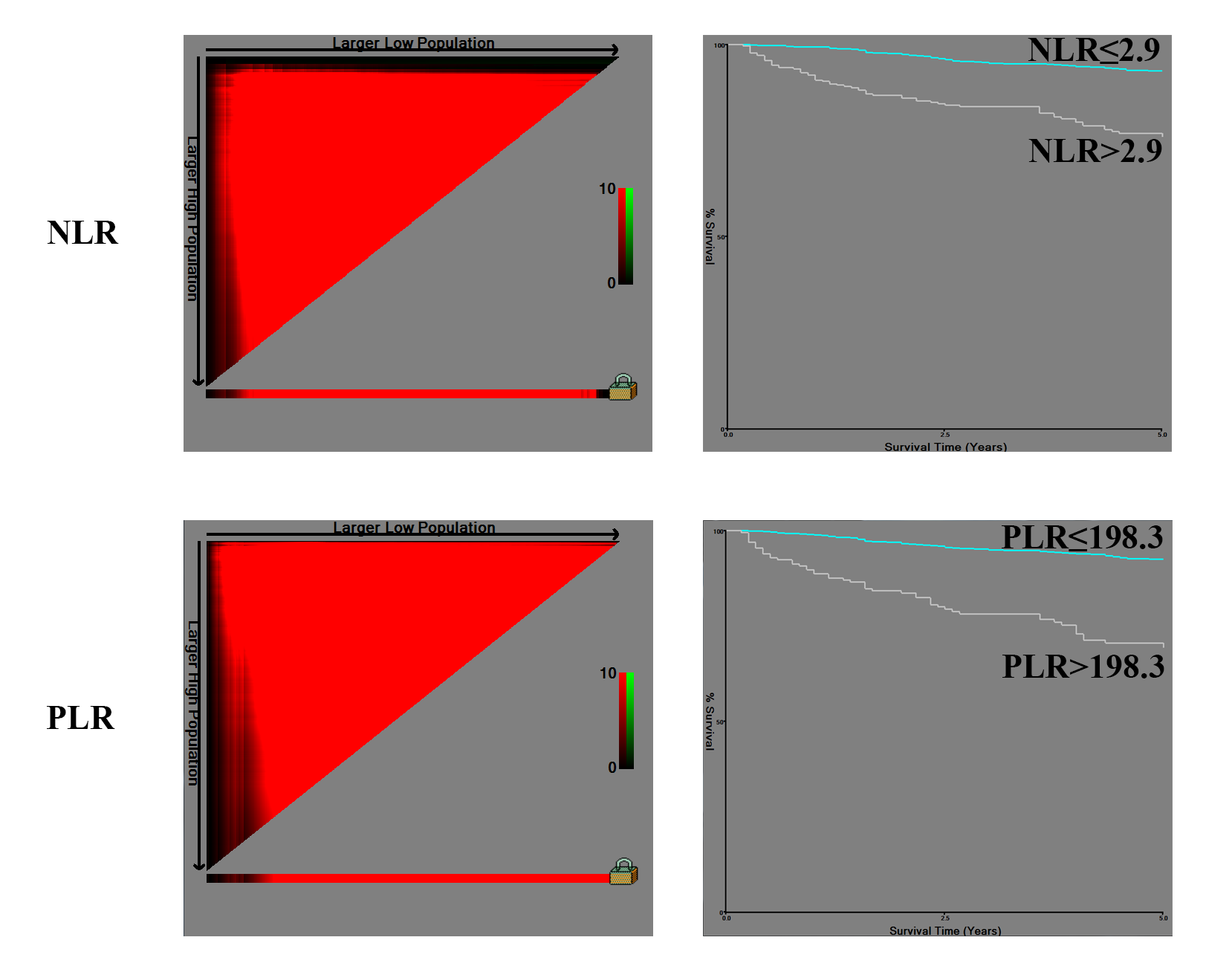

Supplement: Supplementary file 1 — Figure S1. Cut-off values for neutrophil-to-lymphocyte ratio (NLR) and platelet-to-lymphocyte ratio (PLR). (TIFF 6359 kb) [file 12894_2018_333_MOESM1_ESM.tif]
